# Kinaesthetic empathy through the lens of the cinematographer: physiological and phenomenological alignments in the act of creation

**DOI:** 10.3389/fnins.2025.1613485

**Published:** 2025-08-13

**Authors:** William Primett, Ksenia Mukhina, Mehmet Burak Yilmaz, Willeke Rietdijk, Pia Tikka

**Affiliations:** ^1^Baltic Film, Media, Arts and Communication School, Tallinn University, Tallinn, Estonia; ^2^Social Sciences, Institute for Globally Distributed Open Research and Education (IGDORE), Stockholm, Sweden

**Keywords:** affective computing, enactive simulation, physiological data, camera movements, cinematographer's embodiment, kinaesthetic empathy

## Abstract

Film and actor-driven narratives showcase a structured and authentic depiction of emotions, and are considered a reliable resource for validating affective states when coupled with physiological data. In affective computing studies, emotional engagement is often portrayed and perceived as a single-directional mode of interaction between the viewer and the elicitation material. We design a study from the perspective of the cinematographer, who is actively engaged in the creation of the source material while witnessing it. Thus, the image emerges in the interaction between the cinematographer “reading” the actors during the filming, and visa-versa. The captured movement, physiological data, and first-person accounts are gathered from six participants and organized into a multimodal dataset. From this, we introduce a phenomenological data explorer framework, a visualization tool that's used to assess the affective and motor responses during the filming process, while considering the phenomenological experience and narrative immediacy. In turn, we draw triangulations between visual perception, sensorimotor patterns and narrative structures to inform the bidirectional nature of empathic engagement in cinematic storytelling. Our findings emphasize the role of kinaesthetic empathy, in which embodied decision-making responds to predicted inner states, shaping and modulating emotional understanding throughout the filming process.

## 1 Introduction

Dramatized narratives have been shown to induce shared behavioral and neural patterns across different test viewers in the field of affective computing (Picard, 2000; Shu et al., 2018; Fernández-Aguilar et al., 2019; Savard et al., 2024) and in cognitive neuroscience (for reviews see (Hasson and Honey, 2012; Saarimäki et al., 2016; Jääskeläinen et al., 2021). Such physiological similarities may emerge from the viewer's socio-emotional simulation of the situatedness of the fictional protagonists, fundamentally embedded in the broader domain of enculturated conventions of storytelling (Tikka, 2006, 2008, 2022; Deterding et al., 2017; Grodal, 2009; Fingerhut, 2021; Hven, 2022). As such, neural synchrony metrics, derived from physiological patterns, have been studied to examine shared experiences in the the presence of film narrative (Pérez et al., 2021; Kaltwasser et al., 2019; Cohen and Parra, 2016; Dmochowski et al., 2012). While new knowledge has been accumulated on film viewers' experiences, less is known about the experiential nature of filmmaking, our focus in this article. Specifically, we lend attention to the affective embodied behavior and physiological responses that can be observed during the creation process of an acting cinematographer, responsible for capturing dramatized scenes through chosen camera angle and movements in a way that aligns with the emotional situatedness of the actors.

We draw support from the theory of enactive mind (Varela et al., 1991), which assumes inseparable interrelation between the body, the brain and the world. This approach describes an enactive organism in terms of sense-making, embodiment, emergence, and experience (De Jaegher and Di Paolo, 2007, p.487). The holistic brain-body-world system is assumed to rely on moment-by-moment unfolding and largely autonomous predictive processes which are dynamically updated by prediction errors based on feedback from interaction with the world, and validated via interoceptive state of the body; in other words, the brain's predictive process acts as a continuously evolving filter, refined according sensory inputs in comparison with internal models in order to regulate a body in the world. The role of predictive functioning can be conceived within the framework of the free-energy principle, which interprets anticipatory neural responses through the use of Bayesian inference systems (Friston, 2010).

The notion of enactive simulation (Tikka, 2022) is here applied to describe how the cinematographer's brain-body system “lives-by” the protagonist's psychological and emotional situatedness. In other words, the cinematographer's embodied, affective and cognitive processes support the understanding of the protagonists' experience, be that despair, happiness, or social shame. Furthermore, while the cinematographer's perspective (through the camera's viewfinder) to the unfolding of dynamical events between the actors of the scene is considered that of the first-person, the image captured in real time simultaneously embeds the first-person perspective of the prospective viewer (Tikka et al., 2021; Tikka, 2022; Yilmaz et al., 2023).

**Figure 1:**
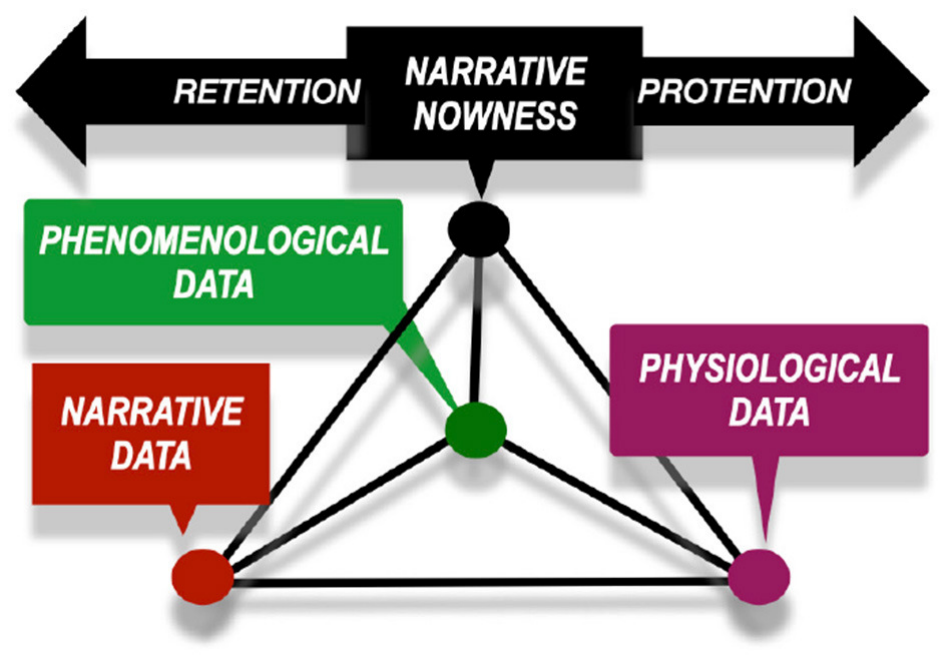
Narrative nowness model adapted from (Kauttonen et al. 2014) assumes that first-person experiences emerge in the dynamical interplay of predictive processes (protention) based on the previous experiences (retention). When collecting synchronous data from physiological, phenomenological and contextual (narrative) data, novel insights to the moment-by-moment unfolding of the first-person experience can be generated.

In line with the *neurophenomenological research program* (Varela, 1996) we combine the quantitative methods from natural sciences with phenomenological methods in order to promote the holistic view to the study of individual lived experiences. It follows that during cinematographer's creative processes phenomenological, physiological and narrative contexts are assumed interdependent and dynamically interrelated (Figure 1) and thus all multidisciplinary data collected and synchronized on the same timeline are assumed intrinsically interrelated (Kauttonen et al., 2014). Regarding cinematographer's creative process of shooting a film scene, on one hand, the first-person experiences unfolding during the moment-by-moment image framing are difficult to record in real time and thus need to be returned to retrospectively. On the other hand, all physiological data as well as the audiovisual data can be recorded in real-time. The challenge remains, how to describe these interrelations and their interplay in the creative processes that dynamically unfold, and adapt time point per time point ad infinitum.

Our aim is to outline a novel approach for phenomenologically and physiologically based experiential assessment whereby the subject is directly engaging with the elicitation material as part of the creation process, allowing their own inferences to influence its narrative qualities. Taking this, we address the following research questions: First **RQ1**, what autonomic and somatic responses can be observed from the cinematographer when engaged in the recording of the actor's performance during a fictional dramatic scene? And second **RQ2**, how does the phenomenological interview data support the analysis of physiological data, in terms of the experiential aspects of the cinematographer?

## 2 Background

### 2.1 Cognitive, emotional, and kinaesthetic empathy

An extensive body of literature on human social behavior is related to the concept of empathy, with “virtual consensus that the empathizer (1) understands, (2) feels, and (3) shares the other person's feelings (4) with self-other differentiation” (Håkansson Eklund and Summer Meranius, 2021, p.302). Here we will deliberately focus on the cinematographer's experience of empathy as a holistic embodied practice (Gallagher, 2001; Colombetti and Thompson, 2008). In line with the 5E framework that considers empathy as embodied, enacted, embedded, emotional and extended (Troncoso et al., 2023), we understand empathy as a complex and multifaceted phenomenon that is deeply rooted in the body and its interactions with the world; an interbodily, interactive process. The proponents of the 5E framework posit that when we sense another person, it is a direct experience as an embodied cognitive agent in a shared context, where bodily/affective processes are intertwined with cognitive processes. This suggests that we may experience the other directly without conscious theoretical inferences or self-simulation (Colombetti, 2014). Our bodies respond and resonate with the movements, postures, and affective states of others at both physiological and subjective levels, creating inter-bodily resonance (Troncoso et al., 2023).

In the domain of cognitive psychology the established terms of cognitive empathy and affective empathy (Shamay-Tsoory, 2011) may be better understood in terms of graded empathy with sensitivity to different experiential aspects of social and contextual situatedness (Levy and Bader 2020). In the literature, the cognitive aspect of empathy typically refers to the understanding of another person's thoughts, intentions and expressed emotions in observed context via mentalizing, described also as the Theory of Mind (Frith and Frith, 2005). In turn, affective empathy is described as getting involved with another person's perceived emotions to such extent that the observed situatedness of the other elicits bodily, visceral response in the perceiver's own body, the process associated with automated neural mirroring, or embodied simulation (Gallese, 2005). To what extent these psychophysiological dimensions can be separated and labeled as they emerge within a social interaction continues a challenge due to the differences in personality traits, biases and context. For example, using fictional drama films as stimuli in their neuroimaging study Raz and Hendler (2014) showed that cognitive empathy and affective empathy can be related to the different temporally defined contexts in the film, affective empathy induced when the protagonist's tragic fate (e.g. death) is taking place on the screen at that very moment of viewing, while cognitive empathy was associated with the protagonist's tragic death (e.g. cancer) to take place in the future (Raz and Hendler, 2014).

By introducing the additional notion of kinaesthetic empathy, we interlink specifically the embodiment of cinematographer's cognitive and affective responses with the bodily dynamics related to sensorimotor movement control, proprioception, and spatial awareness of the actors' situatedness. While practices related to kinaesthetic creativity (Hsueh et al., 2019) and awareness (Françoise et al., 2017) lend attention to a first-person somatic account (e.g. Núñez-Pacheco and Loke, 2022; Höåk et al., 2018, kinaesthetic empathy extends this framing into the domain of embodied intersubjectivity (Di Paolo and De Jaegher, 2015), where affective connections arise between participants who are able to read, decode and respond to the movements of others (Fogtmann et al., 2008; Reynolds and Reason, 2012; Hsueh et al., 2019). Kinaesthetic empathy has been practiced as a choreographic method to study the aesthetic perception of movement and motor simulation (Kim, 2015). The practice has also been applied in Dance Movement Psychotherapy (DMP) practices through movement mirroring exercises (Rova, 2017; Sheets-Johnstone, 2010), where the exchange of gestures, postures, and rhythms facilitates an emotional dialogue, distinguishing it from mere mimicry by emphasizing emotional resonance and empathic engagement (Ehrenberg and Wood, 2011). Kineasthetic empathy may be further interlinked with the discussion on the embodied metaphors (Lakoff and Johnson, 1980) as well as audiovisual metaphors and embodied image schemas (Coëgnarts and Kravanja, 2012; Fahlenbrach, 2018). Here, such kinaesthetic empathy is assumed to take place between the cinematographer and the actors during filming, realized as a bidirectional, dialogical bodily interaction grounded in a type of somatic awareness of each others' situatedness, reflected in the camera movements, resulting in a contextually justified framing of captured images.

When a cinematographer engages in the task of recording of actors' performance with a camera, it is assumed to rely on the generative predictive processes in the brain that not only model the world based on anticipated external events and related sensory input (exteroception) but also via interoception (Craig, 2002), this is, similar type of prediction assumed to take place in “the body's internal milieu (i.e., autonomic visceral and vascular function, neuroendocrine fluctuations, and neuroimmune function)” (cited Kleckner et al., 2017; see also Allen and Friston, 2018; Hutchinson and Barrett, 2019).

### 2.2 Computational modeling for affect and lived experience

The challenge we are tackling in this paper is that, while general physiological and affective measures exist for dramatized narratives, they are rarely applied in real-time creative settings that would allow the creators' agency to emerge through the actors' situatedness in the scene. Existing research often overlooks the dynamic, first-person bodily experiences unfolding over time. Insights from the affective computing field on performative interaction dynamics may provide plausible solutions for measuring the kinesthetic empathy that emerges between actors and cinematographers. In addition, there is a growing shift from traditional quantitative approaches of emotion modeling to implementing more nuanced qualitative techniques that capture the complexities of lived experiences as addressed by Boehner et al. (2007); Bardzell (2010); Loke and Schiphorst (2018), supported by phenomenological descriptions [e.g. micro-phenomenological interviews (Prpa et al., 2020)]. We assume that phenomenological first-person reflections of the cinematographers in regarding their retrospective accounts, this is (in part), elicited interoception, may further be correlated with and described in terms of affective dimensions of arousal and valence; “all psychological events exist in affective space” (Hutchinson and Barrett, 2019, p.8).

The neural and physiological changes related to emotional responses to the fictional dramatized scenes may be observed in the coherence and specificity of the changes in the sympathetic and parasympathetic nervous systems comprising the Autonomic Nervous System (ANS) (Levenson, 2014). Following Levenson's account for confirming coherence, three criteria are to be met: (a) subjective, behavioral, and physiological responses need to be measured continuously over time within individuals; (b) different temporal characteristics of various response systems need to be accounted for; and (c) levels of coherence must be measured when subjects are actually in the throes of a strong emotional experience” (Levenson, 2014). Regarding the specificity, Levenson argues for basic emotions, identified from e.g. the facial, bodily, and vocal signs. Here, we however rely on Lisa Feldman Barrett's theory of constructed emotions (Barrett, 2016; Hoemann and Feldman Barrett, 2019), which allows assuming that affective states can be traced in arousal and valence dimension emerging within the descriptive specificity of the experiential context. A key challenge is that physiological changes detected in situations where the participants are observing other people acting, such as film viewing, may not reflect those in interactive, emotionally contextualized settings (Cole and Gillies, 2022), as in such settings, emotions are shaped by personal involvement and lived experience. The interactional perspective calls for adapting scientific methods to capture these first-person, real-world dynamics (Hååk et al., 2008; Bhanot et al., 2020; Dorneles et al., 2023). Moreover, context-awareness significantly influences how emotions are categorized by the test persons (Hoemann et al., 2020; Dorneles et al., 2023).

Physiological signals have been studied as a resource to uncover emotional exchanges on an interpersonal level. Aly et al. (2024) recorded physiological signals from theater actors who were instructed to use mental imagery to produce specific types of experiential situations and act emotions as-if living those situations in real-time in front of the live audience. Bota et al. (2023) showed that group dynamics play a significant role in emotion recognition by measuring interpersonal similarities in physiological responses during conversations and video-watching. Contemporary stage performances have integrated wearable devices, enabling real-time feedback between the performers' physiological activity and the audience (Françoise et al., 2017; Giomi, 2020; Aly et al., 2021; Zhou et al., 2021; Naccarato and MacCallum, 2016). While being exposed to the performer's inner state, the traditional account of the beholder's involvement (Kandel, 2012) is modified via interactive co-creation through proactive sense-making and affective decoding (Fedorova, 2020; Gemeinboeck and Saunders, 2017). In context of the spectator-performer relationship in live performances, the terms “inner mimicry” and “kinesthetic sympathy” have been used to describe how spectators anticipate physiological changes of the dancer, allowing spectators to connect with and access dancers' emotional experiences (Reynolds and Reason, 2012).

In the case of an active filming process wherein the cinematic imagery is coming into being through the camera work, such beholder's involvement (elsewhere typically related to the viewer of an artwork) can be transferred to describe the enactive simulation process first taking place in the mind of a cinematographer, whose eye is glued to the camera' viewfinder while recording the actors' performance unfolding on the film set, and secondly, to the mind of a viewer when the scene plays out on the cinema screen. In the following we study such processes in terms of phenomenological first-person accounts, measured physiological signals, and observed bodily movement patterns in space and in terms of the actors' emotional and spatial situatedness.

## 3 Materials and methods

### 3.1 Scene, environment, and participants

We examined cinematographers' creative processes, especially focusing on how cinematographers convey meaning through handheld camera movements while filming a dramatized scene in a controlled studio setting. The scene is based on an original screenplay for fictional drama (devised by the 3rd and 5th authors), taking place in one location. The scene featured two professional actors playing a couple: the female character storms into a kitchen in frustration, and is followed by the male character, who tries to persuade her to return to their party in a dominant manner. She resists, claiming his friends dislike her, and the scene ends ambiguously as they embrace. The actors maintained minimal expressions and repeated the scene consistently across eight takes per cinematographer (six in total). The scene's dramaturgy, shaped in part by temporal shifts, is further explored in our results, Section 4.1. An access link to a sample recording can be found under the Data Availability Statement at the end of the article. For our analysis, the final film was rendered at 24 frames per second, with a resolution of 2, 048 × 1, 152 pixels and a 16:9 aspect ratio.

The study was set up by a group of professional filmmakers and was situated in a fully equipped film studio, where the aspects of production quality were maintained to a professional standard to align with authentic experiences of filmmaking. In this sense, the studio setup replicated professional film production, with dramatic lighting and a high-end cinema camera ensuring a cinematic experience. The study participants agreed to fulfill the role of directors of photography (DoP), responsible for the visual composition and camera operation. The primary light sources were emitted from the two windows located on adjacent walls, supplemented by ambient light from the ceiling, each diffused with translucent materials. This lighting configuration provides clear visibility to the actors' expressions while reinforcing the melancholic atmosphere of the scene. Audio was recorded using a shotgun microphone suspended above the actors, which would capture the spoken dialogue in addition to audible movements, such as footsteps, the dropping of the shoes, and dynamics of physical contact (grasping the other, or a sudden embrace).

Six professional cinematographers were recruited for the study, relying upon a convenience sample from the researchers' professional networks to take part on a volunteer basis. The participants all male, between 30 to 40 years old, held at least ten years of experience in cinematography, and had an established professional career in filmmaking. All participants gave their written consent to participate in the study after being informed about the procedure and the duration of the experiment. No risk factors listed in the Ethics Committee of Tallinn University guidelines were present during the experiment. Each participant was compensated with movie tickets to their local theater for their time.

### 3.2 Task procedure

Before starting the filming session, the participants reviewed an information sheet and provided written consent, confirming their understanding of the study and their rights. After signing the consent form, the participant was equipped with the wearable sensors, and the camera ergonomics were tested to ensure comfortable operating. Cinematographers were not given specific instructions beforehand regarding how they should capture the scene, ensuring their responses were guided by implicit expertise derived from their professional experience, rather than predetermined plans. A set of general guidelines were provided as follows:

1. Watch the rehearsal without the camera
2. Shoot three takes of the same shot.
  (a) Start each take from where you are now.
  (b) End each take with a tight two-shot.
  (c) In each take you are welcome and encouraged to do small refinements.

In this context, being a study with cinematographers, the tight two-shot at the end of the take refers to keeping both actors within the camera frame while at close proximity. This shot should also ensure that the actors occupy the majority of the frame. The industry standard Arri Amira camera^1^ was used with Zeiss CP.2 prime lenses.^2^ Regarding the peripheral hardware, only the wireless video transmitter, remote focus motor, and mattebox was placed on the camera, maintaining a lightweight setup, and thus comfort during movement.

The cinematographers were asked to film a total of six takes, with the option for one additional take upon special request. These takes were divided into two sessions, each consisting of three consecutive takes, with a break between sessions. For each session, the cinematographer was assigned either task A or task B. Task A involved using a handheld camera to capture the scene from the female character's (SHE) perspective, while task B required capturing the scene from the male character's (HE) perspective, for which we will refer to as She-focused and He-focused takes. The story context was primed identically for both tasks. Tasks were counterbalanced in two orders, three cinematographers for each order: 3 She-focused takes, followed by 3 He-focused takes; and 3 He-focused takes, followed by 3 She-focused takes.

### 3.3 Physiological and motion data

#### 3.3.1 Physiological measures

**Figure 2:**
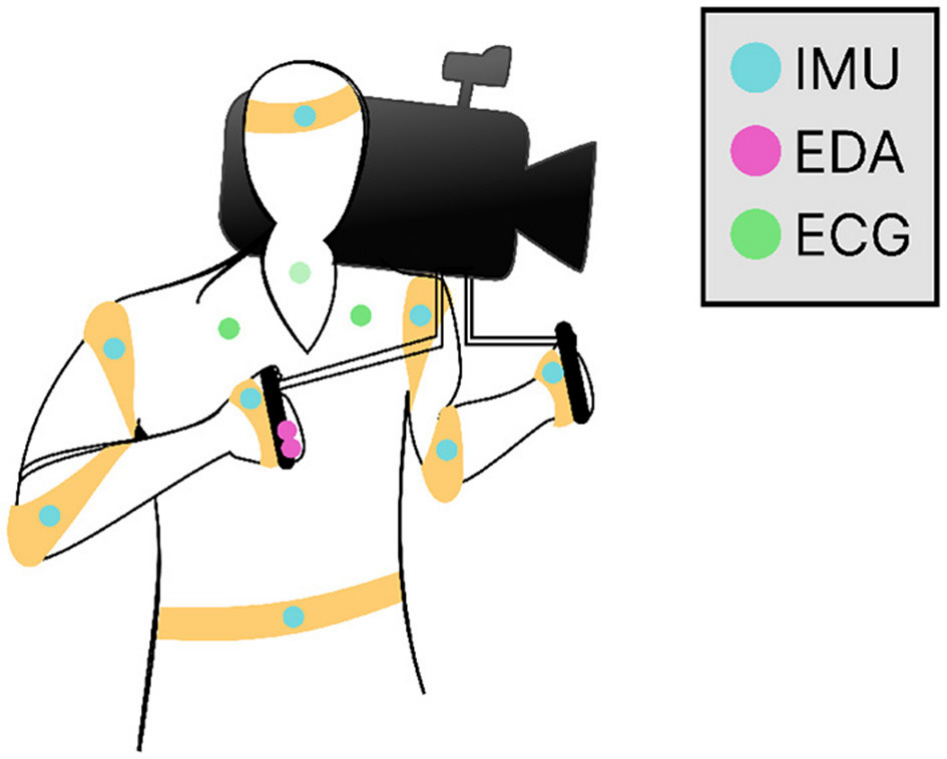
Sensor placement for IMU (cyan), EDA (magenta) and ECG (green) signal acquisition. Two electrodes were placed on the index and middle finger of the subject's right hand to acquire EDA data. For single-channel ECG monitoring, negative and positive electrodes are places on the upper chest, and one ground electrode on the cervical spine.

Electrodermal activity (EDA) and electrocardiogram (ECG) data was recorded to monitor physiological responses during the filming process. These physiological changes can be used to infer information about the subject's inner affective states (Sharma et al., 2019; Schmidt et al., 2018). EDA, reflecting changes in skin conductance, is often linked to emotional arousal and stress, while ECG provides insight into heart rate and variability, which are associated with arousal. By combining these modalities, we can gain a more nuanced understanding of emotional responses in dynamic contexts that involve media engagement and performance-based tasks (Bota et al., 2023). For EDA measurements, we positioned two Ag/AgCL gel electrodes around the center of the subject's right index and middle fingers, ensuring stable measurements while minimally interfering with their hold on the camera's support arms. For single-channel ECG monitoring, negative and positive electrodes were placed on the upper chest, and one ground electrode on the cervical spine. Eight inertial measuring units were strapped around the upper body of the cinematographer to measure acceleration of the limbs (although we do not report on this data in our results). Sensor placements are illustrated in Figure 2. The motivation for using these modalities comes down to the ability to capture stable signals during high-intensity movements, while being less invasive to the task. This is informed by our own tests and the taxonomy of common sensing modalities from (Da Silva, 2017).

The raw data was acquired using an array of Bitalino R-IoT devices.^3^ The choice for using this device was informed by previous studies successfully acquiring data in real-time creative settings (Aly et al., 2024; Primett et al., 2023; Correia et al., 2022). Further, the BITalino ecosystem enables convenient hardware customization for multimodal sensing (e.g EDA, IMU, and PPG simultaneously) within a small form factor that minimizes discomfort and invasion during movement (Plácido da Silva et al., 2014). Additionally, the built-in Wi-Fi based communication protocol was advantageous for long-range data transmission, synchronously with many devices.

A host computer was configured to record data from all devices during the experiment. Afterwards, the signals were synchronized to the video recording and processed using BioSPPy Python library (Bota et al., 2024). Using this library, a built-in bandpass filter was applied to reduce signal artifacts caused by movement of electrodes or electrical interference from filming equipment. From here, the R-peaks, RR intervals and HRV metrics were computed using the Hamilton segmentation algorithm that's included in the BioSPPy library, using the library's default parameters.^4^ For the EDA data, the same library was used to compute the timings of the phasic onsets and peaks, further described in our results, Section 4.3. ECG and EDA data was sampled at a frequency of 250 Hz and 100 Hz respectively. In our data analysis, the physiological data is divided into 13 s windows; this aligns closely with the sequence of narrative phases described in Section 4.1. This time window was sufficient to detect a meaningful number of phasic peaks in the EDA signal, and compute time-domain features of the ECG data, providing HRV metrics for each window throughout the take (Munoz et al., 2015).

#### 3.3.2 Movement tracking from fixed camera recordings

A fixed camera was placed just outside of the main filming area to capture the scene including cinematographer and actors during filming. For each session, the fixed camera was set up to record each take separately starting from the moment the cinematographer takes the position and then continuously recording until the end of the take.

Movement tracking data of cinematographers and the crew was extracted from the fixed camera recording by applying computer vision methods related to object detection, the process of identifying the area of an image or series of video frames where a targeted object is located (as a bounding box or object-shaped segment). Using this method, it is possible to track the cinematographer in motion by combining information about detected objects (here, a cinematographer in motion) from consecutive video frames into a series of positional coordinates in time, defined as the center of the detected bounding box for each frame. A deep learning model YOLOv11 (Jocher et al., 2022) was used to extract initial tracks of cinematographer and two actors from the video with the confidence (conf=0.5) which represents certainty of the model that detected object is a person and intersection over union (iou = 0.35) which corresponds to continuity between the frames. Parts of track that got separated due to the occlusion objects were merged together after a manual check. Due to the significant occlusion between the film crew and cinematographer resulting in a lack of reliable data for 2 of takes (out of the 37 recorded), they were excluded from the analysis. Thus, we obtained 35 movement tracks in total for our analysis, comprising 18 He-focused and 17 She-focused takes.

### 3.4 Phenomenological first-person interviews

Short interviews (~30 minutes) were conducted immediately after filming each perspective. These sessions explored cinematographers' creative processes during the filming of the take, challenges experienced, unexpected moments, dynamics between them and the actors, and contrasts between the two perspectives. With these shorter interviews we aimed to discover specific moments, actions or decisions, as well as shots to be explored in more detail during the micro-phenomenological interviews (MPI) taking place 1-2 weeks after the experiment.

MPIs were conducted with each cinematographer and lasted approximately 1.5 hours. They comprised walking in detail with the cinematographer through their lived experience of one take that, based on their descriptions of moments during the short interview, felt to them as being of particular interest or working especially well. Facilitating their pre-reflective, embodied, affective memory of the shooting of the take [an embodied re-evocation of the experience, see (Vermersch, 2009; Petitmengin, 2006; Depraz et al., 2003; Heimann et al., 2023)] enabled the participating cinematographers to detail their actions, thoughts, feelings, emotions, and sensory experiences during these moments, as stored in their passive and active memory. This re-evocation of their filming of the take under focus was guided by focusing on the sensory characteristics that were present for them and by frequently repeating and summarizing what they shared about their experience of filming the scene, in turn allowing them to provide increasingly fine details about the experience. Full analysis of these long MPIs will be reported on in a separate article; here we present some initial detailed findings regarding key moments of shooting reported by the cinematographers.

#### 3.4.1 Micro-phenomenological interview data

During the initial micro-phenomenological interviews and analysis, phases and sub-phases in the phenomenological unfolding of their filming of the take (the diachrony) were identified for each cinematographer, along with the synchronic data (sensory and other details of experience in each moment) belonging to each phase (Prpa et al., 2020). Before the analysis process, any interview data that does not apply to cinematographers' direct experience but indicates their evaluation, analysis, or generalization of it was discarded, as it is not considered part of the micro-phenomenological data analysis. The original diachrony consists of different phases for each cinematographer, that's part of a full analysis which is not reported on in this paper. For the present paper, a more general synchronic data categorization was performed across all cinematographers, based on the synchronic data belonging to each phase in the diachrony of their filming of the take. Synchronic categories presented in this paper were created inductively by the fourth author, based on the interview data, and remain constant across all DoPs. In the analysis of the 6 interviews, synchronic categories encompass the cognitive, emotional and somatic experience for each significant event. The five categories are as follows: *Thoughts*; *Other mental processes / states*; *Feelings / emotions*; *Body gestures (inner), body sensations, body events*; *Actions (decisive, filming-related)*.

*Thoughts and other mental processes/states* describe how practical decision-making (e.g knowing where an actor was going to move, or focusing on the geometry of the frame) plays a role in the experience, and may reflect cognitive empathy in some cases. *Feelings/emotions* explain what feelings (such as sensing being in sync with the actor) or emotions (such as feeling the emotion of the actor or feeling an emotion about one's one performance) were induced in any given moment of filming, which sometimes shows how cinematographers use their emotional responses to create a frame that induces the same emotional experience for the audience, connected to what they consider the protagonist to be feeling. This category may in some instances relate to affective empathy. *Body gestures (inner), sensations and events* refer to anything sensed or happening in or with the body (for example, inwardly rehearsing one's movements through the set with the camera without actually moving the body awareness of one's body movement and position, feeling the weight of the camera, or physically practicing movements on the set). These provide essential feedback for movement and spatial orientation, and potentially also respond to the actors' inner emotional state. This category might in some cases align with kinaesthetic empathy.

These three mental, emotional and bodily elements intersect as observable *Actions (decisive, filming-related)* made in the moment, which refer to deliberate cinematographic actions such as going closer to the face of the actress, framing the couple deliberately in an unbalanced two shot, moving the camera in sync with the movements of the actor, etc. When these elements intersect, through observable actions, decisions, and filming choices, they collectively contribute to a holistic enactive simulation, which sees bodily events as constitutive of cognitive and affective processes (Colombetti and Thompson, 2008).

As some overlap between these categories is possible, responses that might fall into more than one category were coded in all the categories they were deemed to belong to. Synchronic dimensions emerge within a holistic body-brain-world interaction, and identified and labeled experiential events continue to interrelate with, and embed aspects of other synchronic dimensions. This emphasizes our assumption of a holistic enactive mind situated in temporally unfolding dynamical contexts. In other words, all labels (such as sad, happy, angry, etc.) are tentative constructs to give structure to the lived experience in a specific moment of nowness (synchronic) and during the unfolding of time (diachronic).

### 3.5 Phenomenological data explorer

From the cinematographers' interview data, which consisted of a richly detailed unfolding of events in their filming (such as actors' gestures, choreography, and cinematographers' movements and ways of filming), it was possible to link the diachronic and synchronic data to specific moments of the video of the specific take they referred to, in turn making it possible to connect the micro-phenomenological data precisely with the physiological and movement data on the timeline of that take. The captured movement, physiological data, and first-person accounts are compiled into a multimodal dataset that we navigate using a visualization tool which we call the phenomenological data explorer. This is used to observe synchronized physiological and micro-phenomenological data alongside the individual takes from the experiment, designed to assess affective and motor responses during the act of creation. This framework integrates physiological data with an analysis of the phenomenological experience and narrative immediacy, integrating the integral components contained within (Kauttonen et al. 2014)'s model of experience.

**Figure 3:**
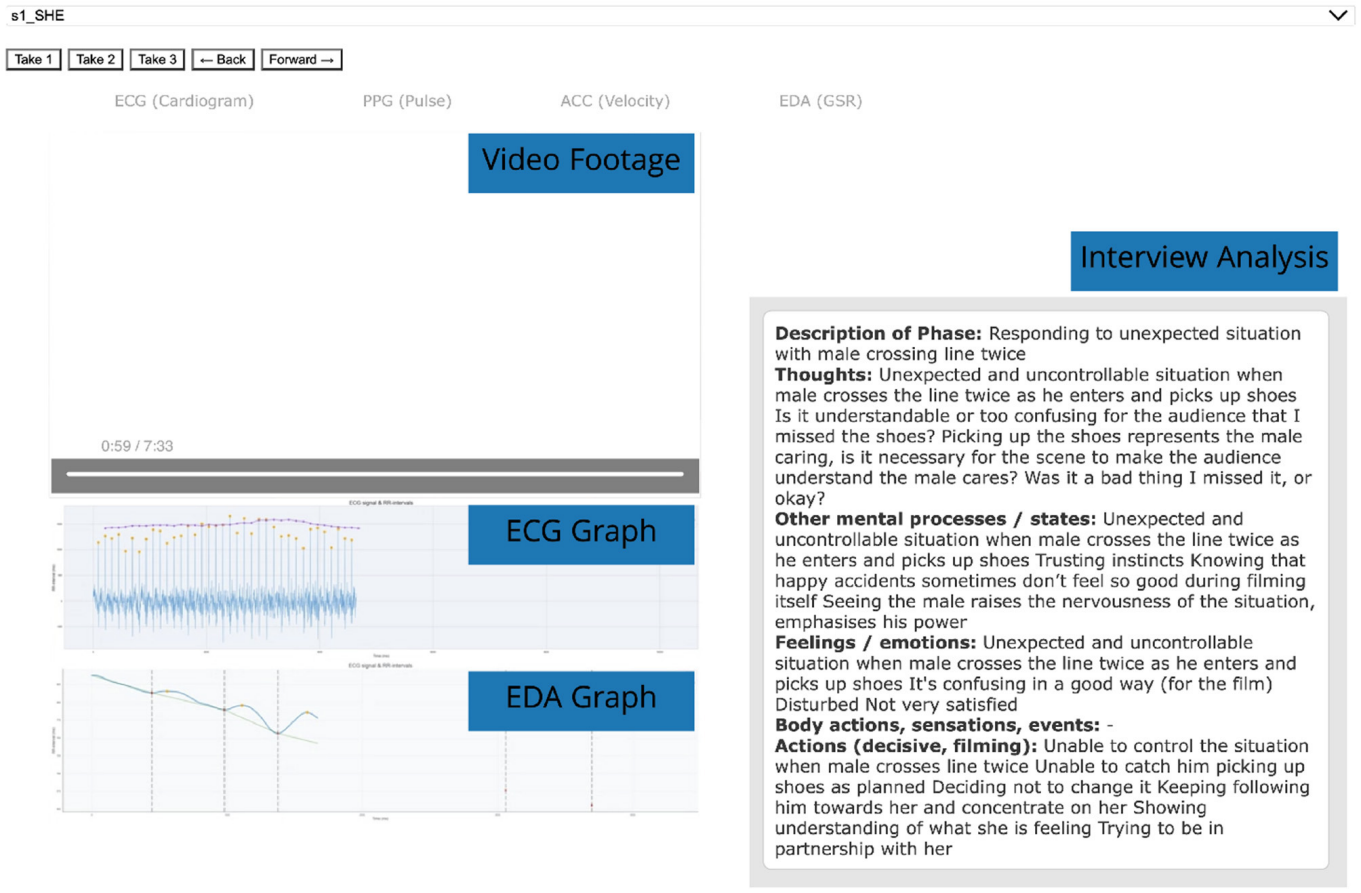
Screen capture of the phenomenological data explorer showing the following components:video footage from cinematographer (top-left), animated graphical representation of physiological sensor data (bottom-left), excerpts from phenomenological interview analysis. The GUI elements facilitate navigation of all the filming sessions and individual takes.

The visualization of the physiological sensor graphs are animated and played back in sync with the video footage based on a shared timecode. By aligning these factors, and focusing on individual events in each scene, the tool enables a precise examination of how the subject's emotional and bodily responses are closely tied to the unfolding narrative moment-by-moment, offering individualized understanding of their empathic engagement. The main components of the phenomenological data explorer are presented in Figure 3. Feature extraction elements are overlaid onto the physiological sensor graphs, essential for a meaningful interpretation. For the EDA data, SCR onset and peaks are overlayed on the animated graph. For the ECG data, the R peaks and RR intervals are displayed, depicting the temporal dynamics of the cinematographer's cardiac activity (heart beat). A closer view of these graphs along with their visualized features and descriptions are presented in our results, Section 4.4.

## 4 Results

In this section, we present a selection of quantitative metrics that characterize the physiological and movement data when analyzed all together on a collective intersubjective level. These metrics provide insight into the autonomic and somatic responses of the cinematographer, addressing our RQ1. This data is mapped to a series of narrative phases that are both relevant and synchronized across all filmed takes. In accordance with our RQ2, we introduce phenomenological interview data to contextualize momentary physiological responses within the cinematographer's own experiential perspective on an individual basis.

We first applied a data driven bottom-up data analysis approach to the acquired physiological data, movement data and the micro-phenomenological analysis of the conducted first-person interviews. The observed patterns of regularities and irregularities in the mentioned data allowed us to apply a cross-referential top-down approach to the other synchronically and diachronically located data sources. For instance, the subjective interview data allowed us to identify meaningful moments and further, based on those moments, to observe the enactive embodied interrelation between the bodily movements of the cinematographer and their physiological responses when engaged in the continuous act of creating the meaningfully unfolding filmic images. Where cited in this paper, the initially pseudonymized cinematographers were assigned new identification codes in a different order from the original one to prevent them identifying each other.

### 4.1 Narrative phases

We identified six narrative key sections and the change points between them based on the sub-events of the story and through observations of the interrelated physiological data, movement data and micro-phenomenological data. All six key phases depicted significant moments that the cinematographers were likely to recognize as events with a beginning, a middle point and an end, indicating a new event starting, and so on. Based on the narrative events of the story, observations of the synchronized physiological sensor graphs, as well as micro-phenomenological reports from the DoPs important narrative transition moments, we were able to examine tentatively synchronized narrative points of both intersubjectively as subjectively relevant experiential moments. The events that define these narrative phases were determined using the data visualization tool described in Section 3.5.

The acted scene begins with her entrance, where she takes off her shoes, stands still, and takes a deep breath before turning to look at the window. Next, in her going to the window, she shifts her focus and moves toward it, with the sequence ending on the word “Darling” as she acknowledges his presence. This leads to his entrance, where he moves quickly into the space, picks up her shoes, places them on the kitchen table, and turns to face her. In the dialogue phase, he approaches her, and the actors face one another while speaking. A key moment occurs when she says “…they don't like me”, which is followed by the initiation of hand-to-body contact, momentarily leading to his hands touching the female actor's face before pulling her close into his arms. The scene concludes in two-shot of the actors in an embrace, capturing their bodily and facial expressions until the cinematographer decides to cut. These six narrative phases span the entire scene from start to finish and are divided as follows (and marked in bold throughout): (1) **She enters**; (2) **Goes to window**; (3) **He enters**; (4) **Dialogue**; (5) **Physical contact**; (6) **Embrace**.

**Figure 4:**
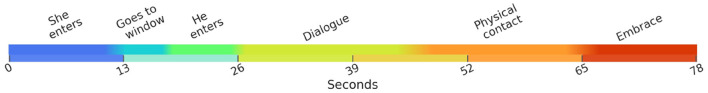
Six narrative phases unfold over the scene's timeline. Average timings of narrative events are color-coded, with fades indicating transitions between phases, averaged colors are shown below for each 13-s sampling window used for time-series analysis, marked on the x-axis.

Throughout our results, we label our time-series data according to the moments that these narrative phases can occur within a set of pre-defined time windows of 13 s, which we refer to as sampling windows. With all the takes, we observe that the same order or narrative phases initiate within each of the given time windows, as the actors maintain consistency across each iteration as they repeat the script. These timings are defined from the average point where the corresponding narrative event begins (listed above), noted from one take for each cinematographer. The first five phases take place during the first 65 s of the film. The length of the final phase is open-ended, determined by the cinematographer's discretion. Some of the phases overlap or conjoin within the 13 s sampling window (visualized in Figure 4). However, in the interest of using a consistent data structure for comparative assessment, we segment the timer-series data into fixed sampling windows (13 s).

### 4.2 Movement patterns of the cinematographers

#### 4.2.1 Normalized time windows and narrative phase alignment

**Figure 5:**
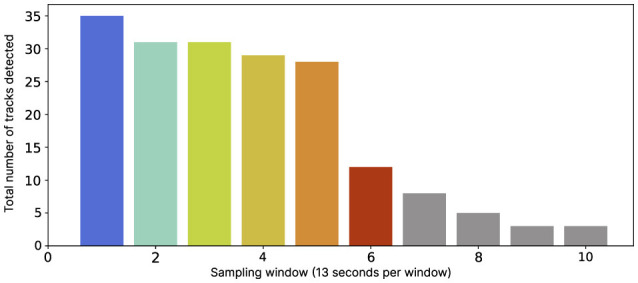
Number of extracted movement tracks per 13 s time window. The graph shows that the majority of movements were detected during the first 78 s (6 windows), before the majority of cinematographers ended their take, with a smaller number of tracks missing due to visual obstruction. Each column represents the 13 s sampling windows that align with the narrative timeline (Figure 4). The blue vertical line followed by gray columns, marks the cut off point for our analysis.

The movement tracks are extracted from the fixed camera footage for the entire take; this provides a broad overview of the movement decisions made throughout the filmmaking process in relation to the timeline of the scene. All cinematographers made their own decisions when to start moving and when to cut the scene (see Figure 5), which results in various duration of different takes. To make up for this fact and also for technical limitations of movement extraction method, we selected 13 s interval which is a minimum time window that assures presences of a detected track for every take of every cinematographer and corresponds to narrative phases of the scene. As can be seen in Figure 5, the majority of cinematographers end their take around 65–78 s, and after the 78 s mark, only a smaller number of cinematographers continued filming additional footage (beyond the original script). For these takes, we limit our analysis up until the 78 s mark, allowing us to observe 6 narrative phases, described in Section 4.1. It is important to note that in some cases, the cinematographers' movements were not fully detected for every phase of every take due to the obstructed view by the film crew. Segments with significant data loss were omitted from our movement data analysis.

#### 4.2.2 Movement data clustering

As part of our data analysis, we apply clustering, a machine learning method, to divide the movement tracks for each cinematographer per take into groups based on their statistical similarity to each other. Since we do not have any prior knowledge of the structure of the data, we compared the results for two clustering methods, K-means and agglomerative hierarchical clustering (Sibson, 1973). Both of these methods are commonly used to cluster time-series data (Radovanovic et al., 2020; Aghabozorgi et al., 2015). To estimate the best number of clusters we used two metrics, silhouette coefficient (Rousseeuw, 1987) and Davies-Bouldin score (Davies and Bouldin, 1979) and tested partitions into a range of clusters between 2 and 20.

**Figure 6:**
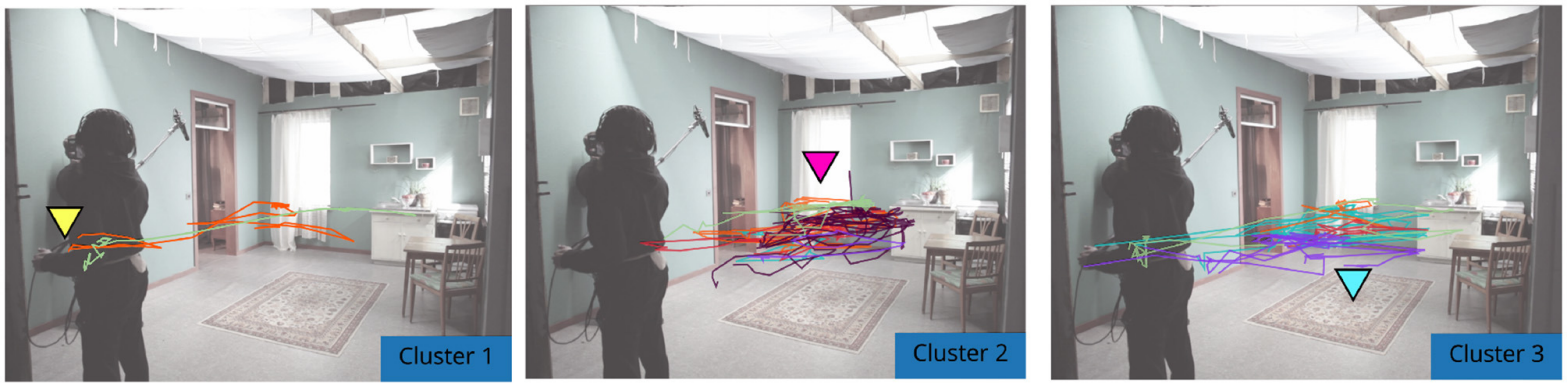
Extracted movement tracks from the fixed camera recording overlaid on initial video frame. The tracks are separated into 3 clusters, each color represents an individual cinematographer. Cluster 1 shows diagonal movement from the origin position (yellow marker) toward the center of the room. Cluster 2 shows movement following the actors toward the window on set, back-left of the room (magenta marker). Cluster 3 shows attention to the location of the shoes dropped onto the floor (cyan marker).

To first identify any reoccurring movement patterns, clustering was initially performed on the movement data captured during the entire take. This clustering revealed 3 general approaches among the cinematographers: cluster 1, diagonal movement directly toward the center of the scene; cluster 2, following actors toward the window in the corner of the room; cluster 3, accent toward the shoes in the center of the scene (see Figure 6). From this, we inferred that the major difference between clusters lies in the decision of cinematographers to focus on a particular part of the scene. To highlight these differences, we split the scene into time-windows and analyse them separately.

#### 4.2.3 Clustering of segmented movement sequences

**Figure 7:**
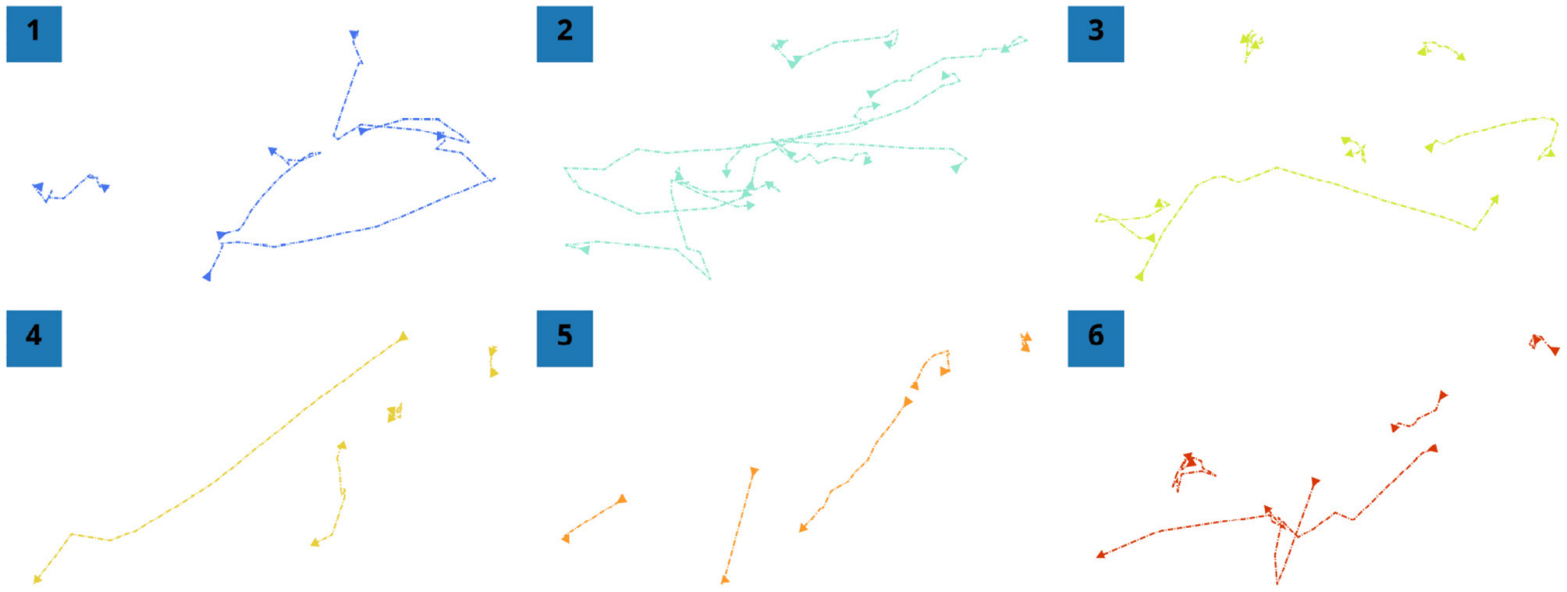
Direction and shape of movement clusters, divided by sampling windows of 13 s. Each line represents the average speed and distance of all movement tracks included in the identified cluster, and the arrow points to the overall direction of those tracks. Individual tracks can be found in Supplementary material.

The optimal numbers of clusters was estimated differently for each sampling window (see Supplementary material for cluster estimation graphs). This indicates that for some phases the scene, the approach taken by the cinematographers was more consistent between their takes, while others spurred more individualistic or experimental approaches. Statistics of obtained clusters, along with their corresponding narrative phases are presented in Table 1. The weighted average entropy is calculated to find the homogeneity of clustered takes according to the he or she-focused task that was assigned (Rohlf, 1974), 1.0 being an absolute separation of tasks, and 0 being an even distribution. The core features that distinguish one cluster from the other are the direction and distance of movement, as illustrated in Figure 7. The distance directly correlates with the speed of movement, given the fixed sampling window for data extraction.

**Table 1:** Description of identified clusters.

**Samplingwindow**	**Narrativephases**	**Timewindow**	**Number ofclusters**	**Averageentropy**
1	She enters	0–13s	4	0.98
2	Goes to window, He enters	13–26s	9	0.83
3	Dialogue	27–39s	6	0.64
4	Dialogue, contact	39–52s	4	0.96
5	Contact	52–65s	5	0.75
6	Embrace	65–78s	5	0.53

Movement tracks are divided into time windows of 13 s, spanning six narrative phases. The # of clusters indicates the amount of groups that were found to exhibit statistically similar movement patterns. The weighted average entropy determines to what degree does the clustering algorithm separate She and He-focused takes for a given phase.

The variation in movement starts from the first narrative phase, **She enters**. In some takes, cinematographers moved toward the window with actors while in others preferred to remain positioned in the middle of the scene. It is worth noting that there is even more distinction between clusters for phases where two actors interact. Thus, for phase 5, **Physical contact**, two clusters (shown top-right) represent filming with little movement from different positions, meanwhile the remaining three clusters illustrate diagonal movements toward different parts of the scene. In addition to that, the first interaction between actors, during the 27–39 s window, the first part of the **Dialogue** phase, lead to the existence of clusters consisting predominantly of one type of he or she-focused takes. The task crossover becomes less homogenous (signified by a higher weighted entropy) for clusters identified in the subsequent sampling window, until reaching the final two phases, **Physical contact**, then **Embrace**. From this, we infer that changing the protagonist's perspective between that of the male and female character influences the cinematographer's choice of movement during these specific segments when the actors are engaging verbally or in close proximity. Given that the weighted average entropy was higher 0.5 for all phases. it can be assumed that variations in the cinematographer's movements were influenced by other factors aside from the set tasks, for which are explored in the following sections of our data analysis.

### 4.3 Physiological activity of cinematographers: electrodermal activity and heart rate variability

**Figure 8:**
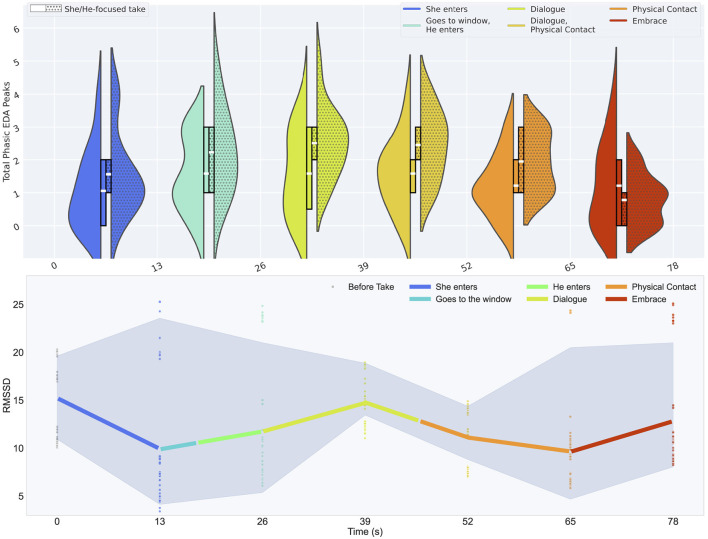
Physiological metrics across six narrative phrases, for 36 takes in total. **Top**: A comparison of average number of EDA peaks amongst all cinematographers, split between takes assigned with She-focused task (left) and takes with the He-focused task (right). The outer violin plots show the overall distribution and the inner box plots show the mean (white) along with the upper-quartile and lower quartile boundaries. **Bottom**: A display of the mean RMSSD metric of HRV, mapped by color. Outliers were filtered using a Z-score threshold of 2.0.

In our physiological assessment, we present electrodermal activity (EDA) and heart rate variability (HRV) metrics on a collective level in relation to the narrative phases. Six takes were considered for each DoP (36 takes in total); any additional takes were excluded from the physiological data analysis. Features of electrodermal activity, particularly phasic peaks, are expected to correspond with emotional arousal and alertness (associated with a “fight-or-flight” response), linked to fluctuations in the sympathetic nervous system (Braithwaite et al., 2013; Menninghaus et al., 2019). For each 13 s window we compute the number of phasic peaks that occur, informed by Hynds et al.'s study on audience engagement in live musical performances (Hynds et al., 2024). For this, we compared the phasic activity of the electrodermal response between the He and She-focused tasks to analyse differences in physiological arousal (Figure 8).

Our findings indicate that the She-focused tasks exhibit fewer peaks throughout the recording, suggesting a lower level of moment-to-moment fluctuations in electrodermal activity. In contrast, the He-focused tasks show a greater number of peaks, with these peaks predominantly occurring during the dialogue, and trailing off until the end of the take. This pattern suggests that the He-focused task may elicit more frequent bursts of autonomic arousal, particularly in response to conversational elements during phase 4, **Dialogue**. For the He-focused takes, we may infer a habituation effect or reduced emotional reactivity toward the end, potentially due to exposure to repeated or evocative affective stimuli in the narrative events leading up to the final phase, **Embrace**. During this phase, the average number of EDA peaks for the He-focused takes drops significantly to 0.78 (with a range from 0.78 to 2.44 peaks overall). In contrast, the EDA peaks for the She-focused takes remain relatively constant throughout, ranging between 1.05 to 1.58 peaks.

**Figure 9:**
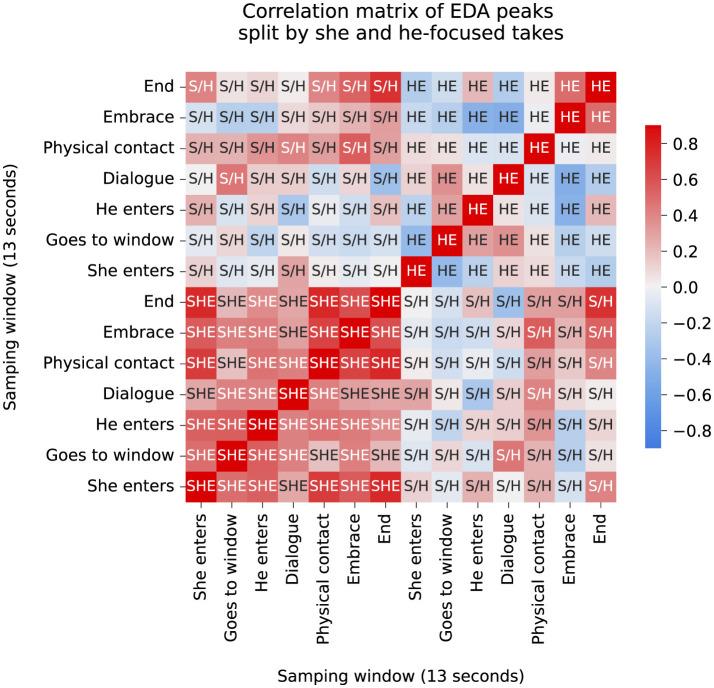
Correlation matrix for the number of EDA peaks for each sampling window. The individual matrix cells reflect correlations computed from 3 she-focused takes or 3 he-focused takes taken by all 6 DoPs. Cells are labeled as SHE, HE, or S/E, according pairwise correlations between the she-focused pairs **(bottom-left)**, he-focused pairs **(top-right)**, or mixed **(top-left and bottom-right)** conditions respectively. For this matrix, we include data recorded after the last narrative phase under the a separate heading (End). When comparing the mean number of peaks across all windows for each participant, a statistically significant difference was calculated between the SHE or HE condition (*t* = 1.83, *p* = 0.08, Cohen's d = 0.61), indicating a reliable intersubject effect for condition-related differences distributed across the entire temporal structure. However, this effect was masked when segmenting the responses into time windows, where a statistically insignificant *p*-value of was computed (*t* = 0.98, *p* = 0.35, Cohen's d = 0.52).

A pairwise correlation matrix is presented in Figure 9, where cells corresponding to the she-focused condition exhibit stronger correlations than the he-focused pairwise correlations across all narrative phases, indicating more consistent EDA responses depending on the cinematographer's attention toward each actor. Linking this with the movement data, we point out that this final phase generally calls focus toward slower micro-movements as the cinematographer and actors arrive a positional halt. To gain further insight into the cognitive and emotional processes of the cinematographer during these events, we refer back to our EDA data throughout our results.

For HRV, we focus on the RMSSD (Root Mean Square of Successive Differences between consecutive heartbeats, derived from the ECG recordings), a metric that reflects parasympathetic (vagal) nervous system activity. An increase in RMSSD is generally associated with relaxation and calm, corresponding to parasympathetic activation and the body's ‘rest-and-digest' mode. Conversely, a sudden decrease in RMSSD signals heightened emotional arousal and excitement, which are tied to sympathetic activation. An accurate calculation of HRV metrics relies on a consistent and clean reading of the ECG data for signal processing in order to provide a valid output. To ensure meaningful analysis, we applied a wavelet-based reconstruction method (MichWozPol/ECG_Denoising, 2025)^5^ and manual outlier removal (Tetelepta, 2025)^6^ to mitigate severe artifacts, which provided us with a stable signal throughout each take, and thus a legitimate representation of HRV, presented in Figure 8. For this, we focus on the physiological patterns shared amongst all subjects, rather than individual characteristics. For instance, a notable point in the HRV data is observed during the moment of physical contact, in which the RMSSD vaslues amongst the cinematographers are most similar, signifying a physiological agreement during this moment.

Since we do not establish a baseline excitement or arousal levels, we focus on the HRV patterns that reflect temporal fluctuations in autonomic arousal, interpreted in relation to concurrent electrodermal activity. During the phases, **Dialogue** and **Physical contact** (39–65 s), there is a drop in the RMSSD (from ~15 to ~10), while simultaneously, the average number of electrodermal peaks increases most predominantly amongst the He-focused takes, and remains at a maximum level across all takes (Figure 8). These concurrent physiological changes suggest a distinct shift in sympathovagal balance, reflecting the relationship between parasympathetic and sympathetic nervous system activity.

### 4.4 Empirical assessment of physiological and micro-phenomenological data during narrative shifts

Illustrative examples of filmed moments are presented below, capturing the dynamics at key transition points where the narrative progresses from one phase to the next. The identification of these change points and their corresponding phases was informed by a systematic analysis of the narrative structure in conjunction with observations derived from physiological measurements, movement data, and micro-phenomenological interviews. This process was facilitated using the phenomenological data explorer tool. At these change points, the combined data shows notable activity or rich descriptions. Seemingly meaningful moments were grouped under phenomenological topics (note that the topics do not represent qualitative themes, as micro-phenomenology data analysis does not comprise a thematic analysis). Below are illustrative examples organized under the corresponding topics. The quotes indicate direct excerpt from the transcribed interviews and replicate the spoken language of the DoPs.

**Figure 10:**
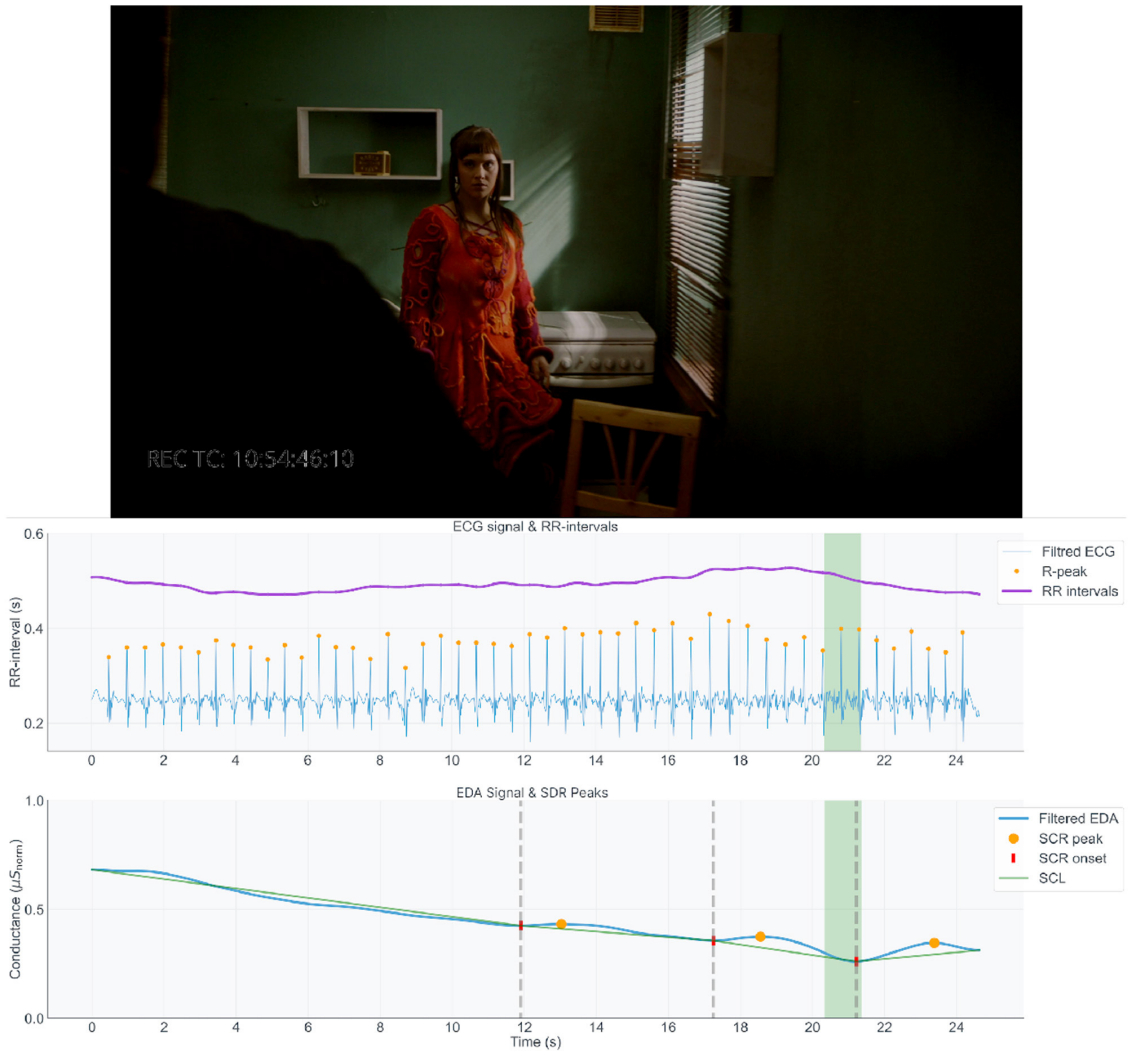
DoP#B filming the moment the male character enters and crosses the line twice in picking up the shoes. The physiological data indicates a decreasing heart rate and an onset in electrodermal activity, highlighted in green.

**Figure 11:**
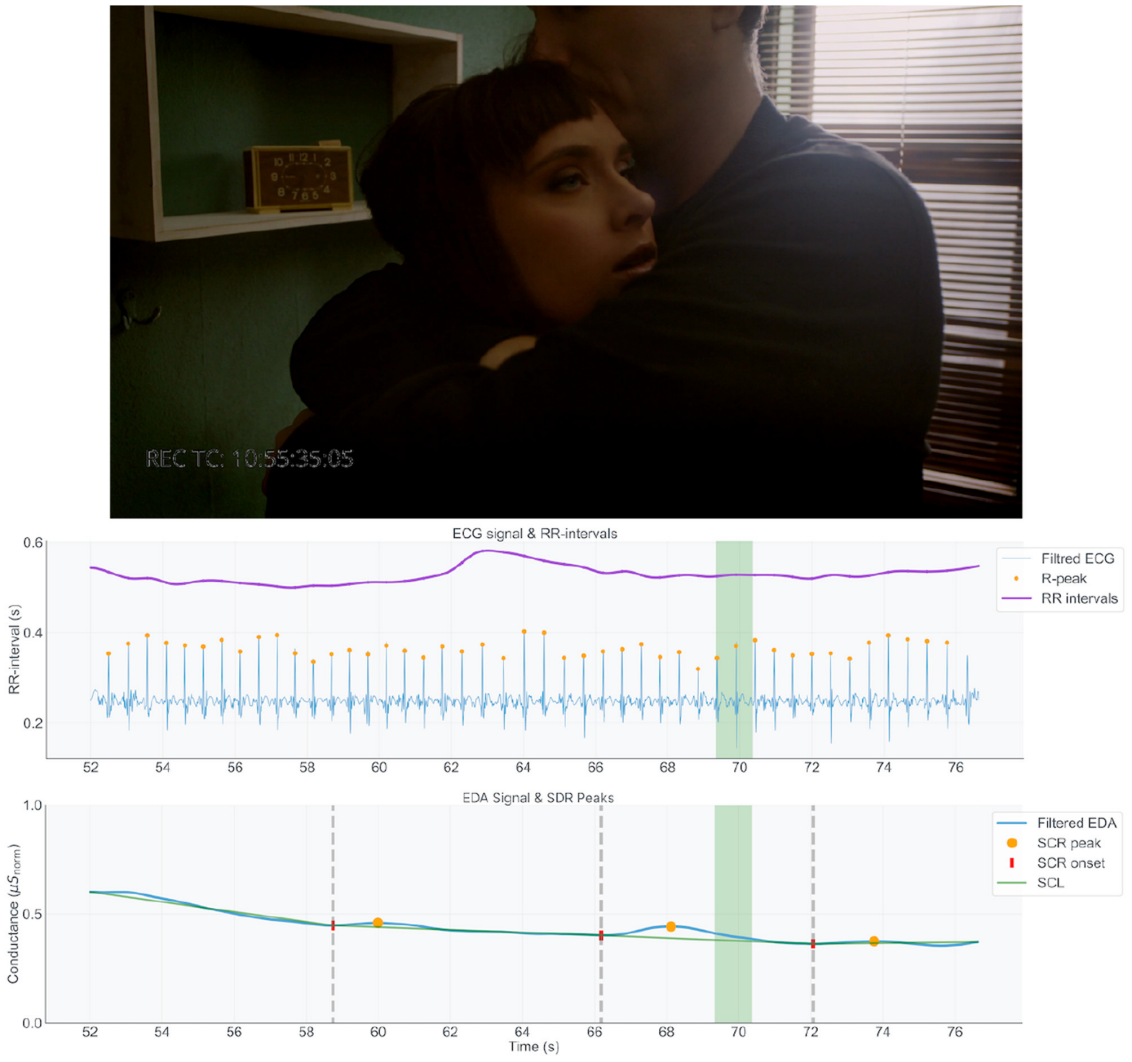
DoP#B Feeling in strong emotional partnership with the female character.

**Figure 12:**
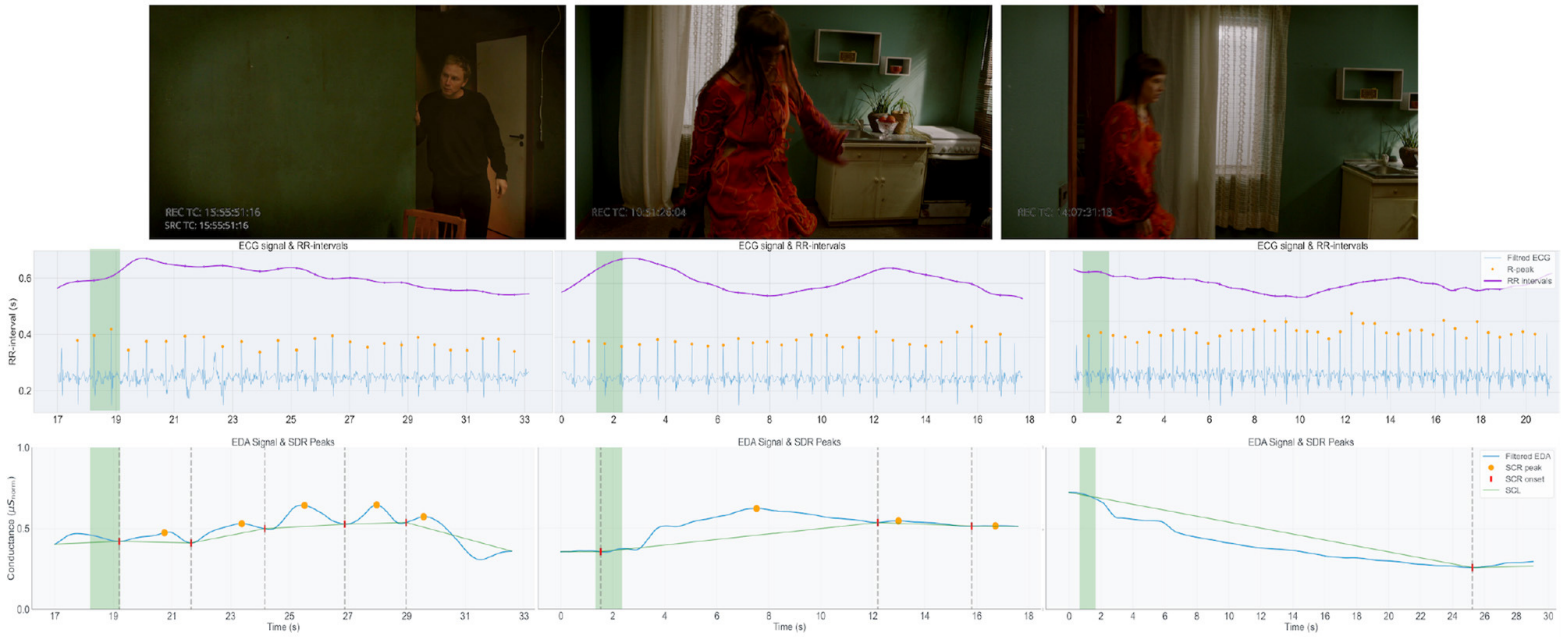
Three examples of feeling in sync with the actors: DoP#F, the “*musical beat”*, which is a blocking point to him (left); DoP#E, locked in tempo with the female actress (middle); DoP#A, feeling “*it is right immediately with this pan”* and feeling connected with the actress from the beginning (right).

The following examples are each presented with a still frame from the captured film, alongside synchronized graphs of the EDA and ECG signals, corresponding to the moment being described. In the EDA signal graph, SCR onsets and peaks are marked in red and orange respectively. The onset-peak couplings are connected with green line that illustrates the SCL trend, separated using vertical dotted lines. For the ECG data, the R peaks and RR intervals are displayed in orange and purple respectively (exemplified in Figures 10–**12**). Graph segments are highlighted to align with the timestamp of the given frame. The visualizations and interview excerpts for the chosen transition points were retrieved using the phenomenological data explorer tool.

#### 4.4.1 Unexpected interpersonal interactions

One of the DoPs described unexpected moments in the choreographic interaction with the actors, which forced him (as well as the actor) to improvise on the spot. Figure 10 presents the 3rd phase, He enters, where DoP#B had to deal with the male character crossing the line twice when picking up the shoes, creating “*an unexpected and uncontrollable situation”* for him. DoP had planned to film the male picking up the shoes, as it showed him being a caring, helping character, but this has now become impossible. “*Was it a bad thing I missed it, or okay?”*, he reports having wondered during shooting, considering the understanding of the story and the male's character's actions by the audience. He decides not to change it and keeps following the male to the female, concentrating on her and showing what she is feeling. He feels disturbed and not very satisfied, but also knows in that moment that what turn out to be “*happy accidents”* don't always feel good during the actual filming. He feels it is confusing in a good way, and trusts his instincts in dealing with the situation.

During this interaction, we observe an onset in the electrodermal activity data corresponding to notable peaks in the skin conductance level, a few seconds after **he enters**, signaled by the initial line, “Darling” (at around 17 s). These peaks are coupled with short recovery times, returning to baseline level. In parallel, the subject's heart rate (RR-interval) begins to gradually reduce during the remaining 4 s of this phase, until reaching a new baseline. From, the physiological activity, we can infer that the actor's movement patterns, deemed unpredictable, induce a degree of stress for the cinematographer. Partially also due to the fact that this “*accident”* happened during the first take of the first task given to him in the experiment.

#### 4.4.2 Reading the character's emotions

Figure 11 shows the moment at the end of phase 6, Embrace, in which DoP#B expresses a strong emotional feeling of partnership with the female character and what she is going through, along with a sense of responsibility to convey this to the audience. The DoP tries to show her emotions (“*like helping her”*) and stays focused on her face and her eyes open toward the ceiling. He feels “*It's working, everything works really well together”*, with all three of them positioned rightly, her having her eye open toward the ceiling, and how the light falls on them. He says, “*now we get to see how she really feels, (…) she is showing me what she is really feeling and thinking, (…) I feel like she is trusting me with a secret. (…) someone is now seeing what she is going through.”*. In the physiological data, we observe a recovery to baseline levels with gradual drop in the RR-interval as well an an onset in electrodermal activity occurring around 4 s prior. The parasympathetic activation (heart rate) alignment with the sympathetic withdrawal (electrodermal activity) points toward a mode of self-regulation that extends beyond basic autonomic recovery, supporting a state of adaptability to engage with the task's demands.

#### 4.4.3 Rhythm: being in sync with actors

Several descriptions were given by the cinematographers of moments in which they felt in sync or deeply connected with the actors, or in which they were filming “*according to the rhythm of the scene”*, or in which the “*rhythm felt right”*, presented in Figure 12.

For example, DoP#F talks about sensing the moment when the male character enters the scene (phase 3) as a musical contrast between silence and articulation. He says “*the male character seems intrigued and excited about the panicky rushing in of the actress. (…) I want to show his emotion, action and reaction. I sense this moment as a musical beat. (…) It seems like a blocking point to me.”* When discussing this moment, the DoP explains that he has “*music, rhythm, and tempo processes going on in the back of my mind,”* signaling that soon he needs to start moving, as the moment is very short. As a result, he is unable to frame the male character in a close-up, “*even though that corresponds with the beat”*. When following the actress closely as she enters the room (phase 1), DoP#E feels the “*rhythm”* is right from the moment she walks in, and that she and the DoP himself subconsciously “*move in tandem, at same pace and mirroring each other”* as they “*lock our tempos”*. We note the RR-interval increasing during these two moments, before reaching a relative maximum, with short bursts of electrodermal activation throughout the first example.

Another example of rhythm comes from DoP#A who starts his last take in the female perspective (she-focused task) by panning from the opposite window to the actress just before she enters (phase 1) to catch her entering. The DoP “*feels immediately that it is right with this pan”*, and starts to feel a deep connection with the actress, which continues for the rest of the take. Perhaps relatedly, he feels the camera (during this take) “*doesn't feel like a character or a point of view; it is not visible”*. Contrasting from the previous examples, the physiological data presents a prolonged return to baseline arousal levels after this moment, potentially reflecting on sustained attention given to the task's executive demands, overriding the emotional reaction to the actor's behavior.

## 5 Discussion

In this study we have provided a multiperspectival window to the professional cinematographers' embodied practices, in the process of shooting a cinematic scene with two actors in a film studio production setting. Our aim has been to observe the creative processes in an ecologically valid situation that is relatively similar to an actual shooting of a fiction film scene. The set up allowed us to record data of the six professional cinematographer's processes in terms of phenomenological first-person accounts, measured physiological signals, and observed bodily movement patterns in space and in terms of the actors' emotional and spatial situatedness.

The three domains of data collection were selected based on the narrative nowness model (Kauttonen et al., 2014) described in Section 1, taking upon the assumption that each cinematographers' creative acts as recorded live (physiological and motion data) or in immediate reference to the lived-by act of doing (interviews), represented some ecologically relevant aspects of their first-person expressions of holistic enactive simulation. In this sense the set-up can be argued to be in agreement with the criteria for coherence argument for psychological (emotional) states by Levenson (2014): “(a) subjective, behavioral, and physiological responses need to be measured continuously over time within individuals; (b) different temporal characteristics of various response systems need to be accounted for; and (c) levels of coherence must be measured when subjects are actually in the throes of a strong emotional experience” (Levenson, 2014).

In line with the idea that affective states are inseparably related to all bio-culturally meaningful and situated experiences, conscious or unconscious (Varela et al., 1991), the first-person interviews allowed us to tentatively identify five categories (1) thoughts, (2) other mental states (e.g. awareness), (3) feelings or emotions, (4) bodily gestures and sensations, as well as (5) deliberate actions related to filming (e.g. frame composition decisions, camera movements, body moments, etc.). Although the detailed micro-phenomenological analysis falls outside of the current paper, the conducted first-person interviews and related initial categories provides contextual information to the physiological and motion data that's used to identify significant changes in the cinematographer's inner state. Below, a selection of the points of interest are discussed in more detail.

### 5.1 Misalignments in cognitive prediction

The holistic embodied perspective we have been promoting in this paper, we assume in line with (Allen and Friston, 2018; Hutchinson and Barrett, 2019) that the core evolutionary survival function for living body-brain-world systems such as humans involve continuous dynamically adaptive exteroceptive and interoceptive predictive processes. A great part of these processes are hidden from the third-person as well as the first-person observation, however, the detective work of us scientists relies on reportable and repeatable observations of humans in action together with computational simulations. The moment in our experiment where the actors unexpectedly looked directly to the camera, an act that the cinematographers were not expecting based on the actors repetitive performances in the previous takes, introduced a moment of surprise, a marked prediction error point for us to observe.

While the cinematographers adapted to the actors' repetitive patterns (according to the task), the unexpected moments in their performances naturally related to the very first takes which could be regarded as rehearsals with camera. For inducing stronger surprise, or a prediction error in terms of (e.g. Hutchinson and Barrett, 2019) to break the repetitive patterns of the acting, at the end of the last (6th) take of each cinematographers' session, the actors were instructed to unexpectedly look into the camera to induce a moment of ‘surprise'. This allowed us to observe how each one of the six DoPs changed their acting patterns along the unexpected behavior of the actors.

These unexpected moments present a novel stimuli which is assumed to trigger a surprise reaction identified as a basic emotional descriptor, characterized by a rapid spike in arousal followed by a fast recovery, returning to a state of equilibrium (inferred from the electrodermal activity). The corresponding physiological and emotional responses are therefore considered to occur momentarily in transients. Our results have partly exposed how the unpredictable elements of the filming process can influence the cinematographer's physiological and phenomenological responses. With this in mind, we also acknowledge that other contextual factors that are inherent to the film production context, such as crew presence, can also affect the emotional resonance and motor behavior beyond what is visible on-screen.

### 5.2 Kinaesthetic empathy as narrative counterpoint

Following Reynolds and Reason (2012) we proposed the term kinaesthetic empathy to describe cinematographer's creative practices through embodied exchange of emotional states with others through dialogic prediction and simulation of the shared movement dynamics, the embodied process that build on moment-by-moment lived engagement with the other persons' affective situatedness. In our view, kinaesthetic empathy revealed its features most clearly in the narrative sections where the sensitivity to observed interpersonal distance and perception of physical contact between the actors became relevant (see details discussed in subsections below). Our analysis of movement tracks shows that even in an environment bounded by experimental constraints there is not a single strategy to approach a creative task. Most cinematographers moved differently from take to take which was shown by the presence of movement tracks in different clusters. Studying shorter movements by narrative phases revealed that direction of the movement, speed and shape of the track itself might significantly differ from take to take even for the fixed scene. During narrative phases where two actors directly interacted with each other (dialogue, contact), the cinematographers generally preferred to limit their movement, supported by the presence of clusters containing very little positional movement.

In everyday social environments Interpersonal Distance (IPD) is defined in silent cues, body language and eye-contact, between interacting persons and varies between people. The acceptable proximity of IPD between two people is influenced “by the expression and understanding of the intentions of the person with whom the individual is interacting” (Huang and Izumi, 2021). While professional actors are trained to work in situations where interpersonal distance may fall into zero, the cinematographers, depending on their personal traits, may be personally sensitive to other people's proximity in everyday life, yet, when working with actors the camera may provide a shield against felt unconformity of closeness. Any discomfort during the camera work may be retrospectively detected in the acquired physiological data, however, only if this is indicated explicitly in the personal interview by the individual cinematographer. The distance between the DoP and the actors is one parameter to be discussed. Another is the observed interpersonal distance between the actors, including their non-verbal bodily cues and perceived physical bodily-contact.

We have identified instances where the cinematographer's movement distinctly responds to the actors in a way that can articulate deep narrative structures. We suggest that kinaesthetic empathy in this context characterizes a multifaceted bidirectional emotional engagement that correlate with enduring cognitive or affective processes, and require experiential accounts to interpret. For this, more nuanced relationship in perceived between physiological response and external stimuli as the body observes a regulatory state (see Figure 11, for example) that's capable of applying predicative functioning, allowing for the timely anticipation of forthcoming events, and their preparation. Further, decisions or feelings characterized by the cinematographer's cognitive engagement, relating to their professional experience, were reflected in prolonged physiological patterns (see Figure 12, for example). The cinematographer reads the character's inner state, and embodies these feelings in perspective of the narrative context, culminating to a narrative counterpoint.

### 5.3 Subjective responses to (un)dramatic events

As expected, while actors repeated the same scene for all the cinematographers at their time relatively similarly in terms of their movement patterns in the space as well as their verbal and bodily expressions, however, several cinematographers reported that they detected modifications and changes in the actors' behaviors between the different takes. Naturally, minor variations in performing the total of 48 (8x6) repetitions were expected as actors are humans and not robots. Recognizing these minor changes may reflect the adaptive processes of the cinematographers in terms of their dynamically updated predictive processes as they perform their task based on the anticipations from the previously completed takes. It was also expected that cinematographers both react on the fly, and retrospectively interpret situations differently from one another in their interviews. Interestingly, the interviews allowed us to identify moments where one's subjective view on the experience of the actor's unforeseen stare into the camera “*didn't surprise me that much in that moment. Or maybe, how to say, translate my surprise into the action of …I reacted, to the surprise.”* (DoP#D), while an autonomic (physiological) elicitation was present in the data, and the surprise moment caused the DoP to move backwards, as it implied actors breaking ‘out' from the scene, *“I think it was a little bit like pushing me back, no? It was like they look at me, and I automatically feel that I have to take distance from them.”* (DoP#D).

## 6 Limitations of the study and future work

The subject pool was limited to the convenient selection of six professional cinematographers with over ten years of experience in the field. In the study we did not plan on relying on averaging between different participants, as their performances were expected to significantly differ from one another due to the designed degree of freedom in the execution of the shooting the task; instead, we expected to collect data from six individuals, each of whom would provide individual insights to the cinematographer's creative processes, thus increasing our understanding of these processes in terms of multiple perspectives and multiple strategies applied to the fulfill the same task. Due to the limited sample size, artifacts in sensor data, and small variants in non-controlled factors such as scene length, we do not anticipate or claim statistical significance as part of our quantitative data analysis, nor do we claim generalizability. Rather, our pilot study results value the relationship drawn between the quantitative data and the detailed first-person accounts that are derived from the micro-phenomenological interviews analysis.

In this paper we focused on discussing kinaesthetic empathy in terms of phenomenological first-person accounts, measured physiological signals, and observed bodily movement patterns in space, in terms of the actors' emotional and spatial situatedness. However, we acknowledge the limitations of the current approach in covering interlinkage of movement control, motor-planning and decision making at the neuro-functional level. Future studies may apply, in addition to the methods used in the current study, wearable electroencephalography for providing insights to the cortical dynamics of cinematographers' action planning, sequential motor execution, predictive processes, as well as reward processes (e.g. Meyer et al., 2021), combined with subjective accounts of first-person experiences after successful completion of the shooting tasks.

## 7 Conclusion

This paper explores the relationship between micro-phenomenological data, physiological data, and motion data to gain a comprehensive understanding of the cinematographer's embodied decision-making in the act of framing the events between two actors with a handheld camera in a professionally valid film studio production setting. This approach linked first-person reports, autonomic nervous system activity, and movement patterns to reveal the connection between subjective experience, emotional state, and bodily actions. We frame these findings in terms of empathy between the cinematographer and the actors, with a focus toward *kinaesthetic empathy*, where bodily responses and movement patterns reflect the inner states and actions of these being observed in the act of creation.

Our micro-phenomenological findings from the present confirmed that cinematographers were able to elicit a number of specific moments during their creative camera work that were significant to them. The analysis of the interviews of the six cinematographers resulted in initial diachronic and synchronic categories that further contributed to the establishment of five narrative phases coordinated with the physiological and movement data. The synchronic categories identified in the interview data were used to detect narrative-dependent experiential moments of felt empathy and connection between the filming cinematographers and actor(s), exemplified by observed interpersonal distance between cinematographers and actors, their reported (de)synchronization with the actors and the scene, as well as their responses to unexpected interactions with the actors.

By situating modes of empathic engagement as an integral function within a holistic enactive simulation, our study addresses the common notion of single-directional emotion elicitation in affective modeling, offering an interactional perspective that integrates the creative process into the assessment of cognitive and physiological responses. This study can offer insight to affective modeling and analysis by highlighting the role of active perception in emotional experiences, providing a novel framework for assessing affect within naturalistic, interactive contexts. We foresee our methodology to be of value in uncovering complex relationships between creation and emotional engagement with other forms of media such as music, theater, and visual art.

## Data Availability

The datasets presented in this study can be found in online repositories. The names of the repository/repositories and accession number(s) can be found below: https://doi.org/10.5281/zenodo.15128617.

## Footnotes

^1^https://www.arri.com/resource/blob/73172/560117a761956ba3141e93c648c4c32d/1-4-0-amira-data.pdf.

^2^https://www.tlu.ee/sites/default/files/Instituudid/BFM/Instituut/Kasutusjuhendid/CompactPrimeLensesCP2_Brochure_EN.pdf.

^3^https://ismm.ircam.fr/riot/.

^4^https://biosppy.readthedocs.io/en/stable/biosppy.signals.html#biosppy-signals-ecg.

^5^MichWozPol/ECG_Denoising (2025). *MichWozPol/ECG_Denoising*.

^6^Tetelepta, S. (2025). *Stetelepta/Exploring*-*Heart-Rate-Variability*.
