# Supplementary material for: Kinaesthetic empathy through the lens of the cinematographer: physiological and phenomenological alignments in the act of creation
Source: Front Neurosci. 2025 Aug 13;19:1613485. doi: 10.3389/fnins.2025.1613485 (PMC12395500; doi:10.3389/fnins.2025.1613485)
Supplement: Supplementary file 1 [file Data_Sheet_1.pdf]

Supplementary Material

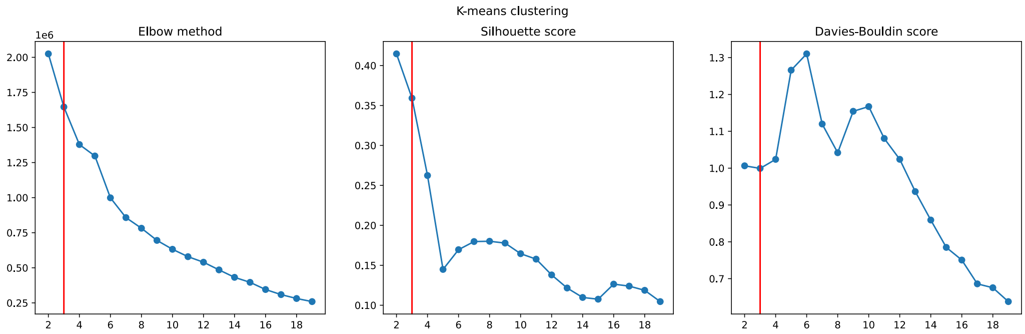

Figure 1a. Silhouette coefficient, Davies-Bouldin score, and cluster distortion for K-means clustering on full movement tracks data.

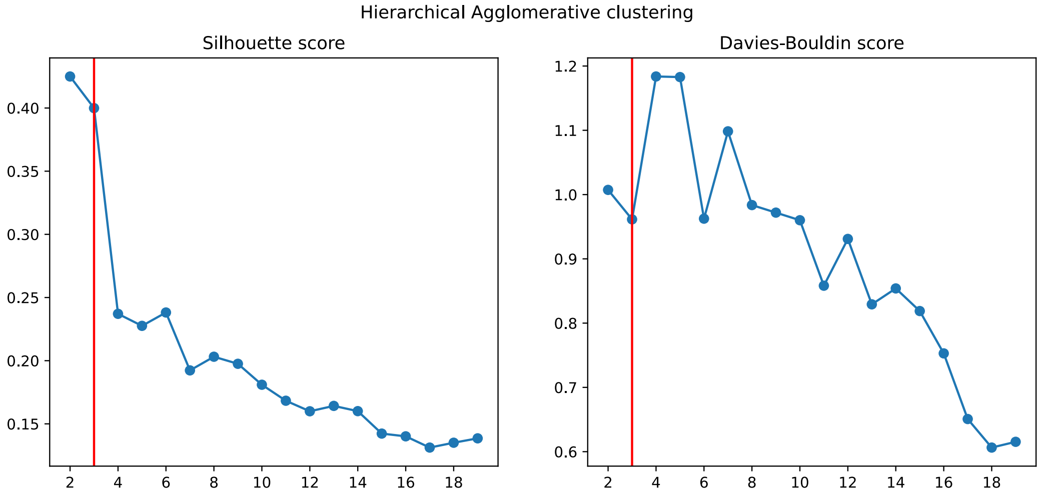

munch

Figure 1b. Silhouette coefficient and Davies-Bouldin score for agglomerative hierarchical clustering.

Figure 1. Identifying the optimal number of clusters using **(A)** K-means and **(B)** hierarchical agglomerative clustering. The number of test clusters varied between 2 and 20. For the silhouette coefficient, optimal number of clusters corresponds to the highest value, meanwhile, for Davies-Bouldin index, the lower the score, the better, and for the elbow method, the number of clusters is identified as the moment of significant drop in cluster distortion. In this case, all metrics for both methods are in agreement that three clusters is the optimal number.

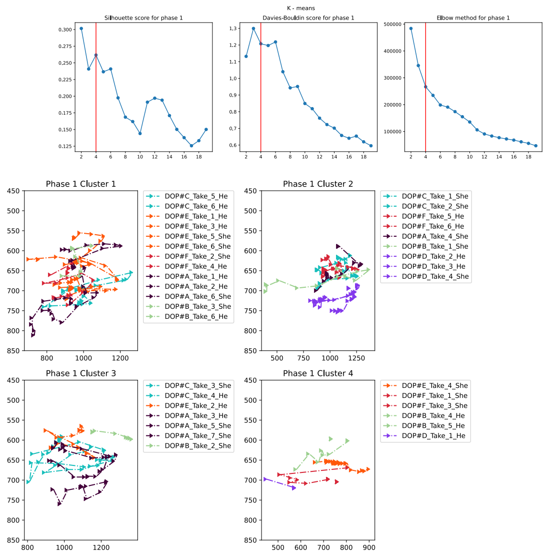

Figure 2a. Narrative phase 1.

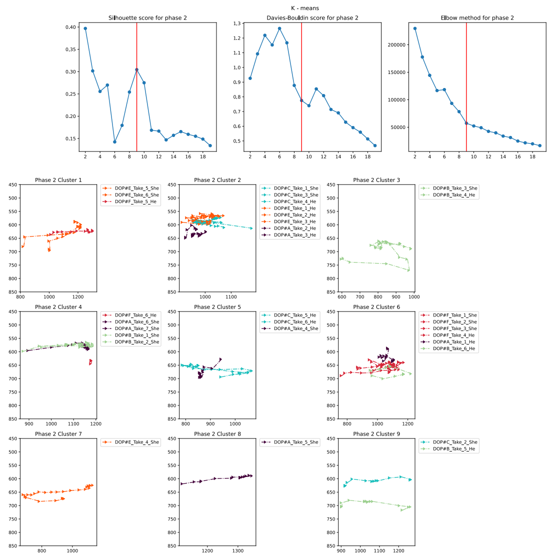

Figure 2b. Narrative phase 2.

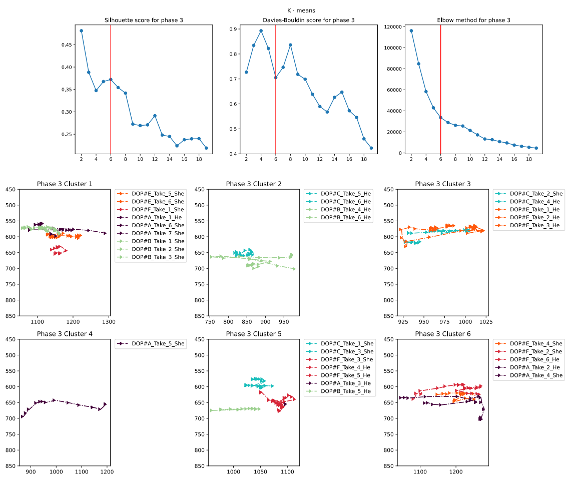

Figure 2c. Narrative phase 3.

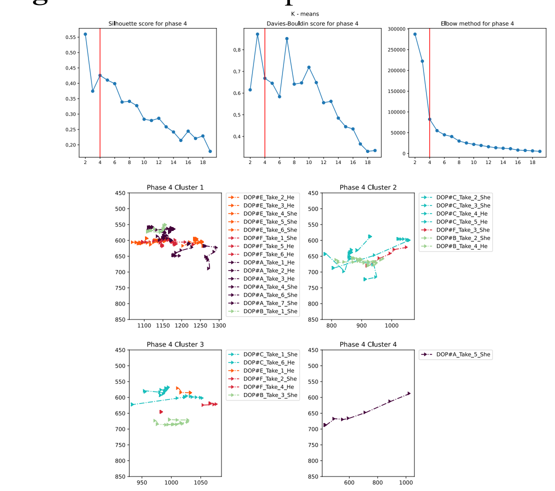

Figure 2d. Narrative phase 4.

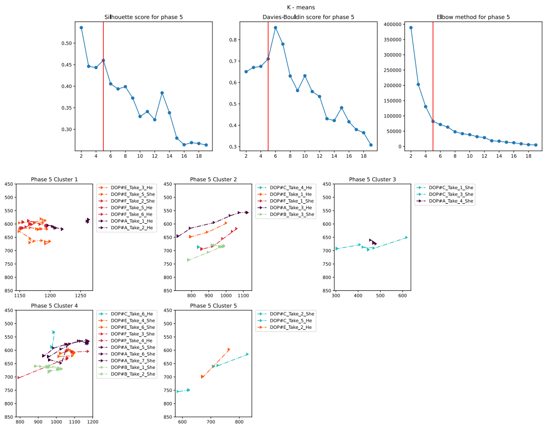

Figure 2d. Narrative phase 5.

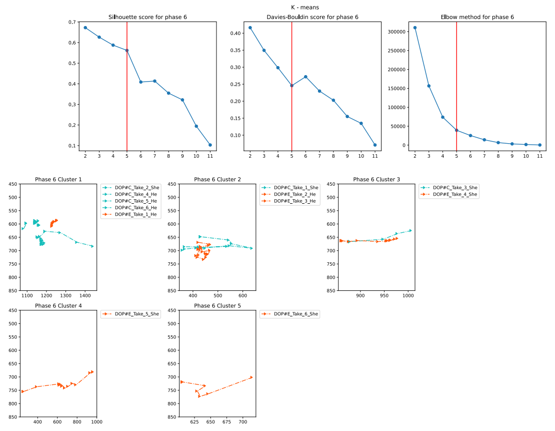

Figure 2e. Narrative phase 6.

Figure 2. Clustering metrics and resulting clusters of extracted movement trajectories for narrative phases **1-6**. Identifying the optimal number of clusters using K-means clustering (top). Following a similar approach, the optimal number of clusters was identified for each narrative phase. In this case, we tested only K-means clustering but used all three metrics to select the result.
